# Vitamin D in Health and Disease 2.0

**DOI:** 10.3390/biomedicines12020324

**Published:** 2024-01-31

**Authors:** Giuseppe Murdaca, Sebastiano Gangemi

**Affiliations:** 1 Allergology and Clinical Immunology Unit, Department of Internal Medicine, San Bartolomeo Hospital, University of Genoa, 19038 Sarzana, Italy; 2 Allergy and Clinical Immunology Unit, Department of Clinical and Experimental Medicine, University of Messina, Via Consolare Valeria, 98125 Messina, Italy; gangemis@unime.it

Vitamin D (VD) is a fat-soluble vitamin considered essential for human health, and its levels are associated with the function and composition of the intestinal microbiome. In fact, it plays a decisive role in the pathogenetic process of immunological diseases related to the intestinal microbiome starting from the pediatric age; its integration is in fact capable of increasing the diversity of the microbiome in children, with a consequent positive impact on the functionality of the immune system. It has been demonstrated that VD is capable of increasing the number and variety of commensal bacteria, which are able to compete with pathogenic bacteria for nutrients, thus exercising a synergistic protective function against various diseases [1]. 

Recent evidence has also highlighted the potential role of VD in allergic and immune-mediated diseases, exerted by its ability to modify the immune response and the proliferation and function of mast cells, particularly through the modulation of the IL-31/IL-33 genes axis [2]. Increasing its concentration within the intestinal bacteria responsible for the release of short-chain fatty acids would increase the secretion of immunoglobin A by B cells, reducing macrophages and dendritic cells and limiting chemotaxis in the neutrophils. All this would confirm the fundamental role of VD in innate and adaptive immunity, in addition to its anti-inflammatory properties. 

Free serum VD is stabilized and transported to target tissues by VD-binding proteins (VDBPs), where VD exerts its biological effect at the cellular level by binding to the VD ligand-activated receptor (VDR), belonging to the superfamily of nuclear receptors. After binding to the biologically active form of VD, VDR enters the nucleus and forms a heterodimer with the retinoid X receptor, thus regulating gene transcription. Several subtle allelic polymorphisms have been identified in the gene encoding VDR and have been linked to diseases such as osteoporosis and metabolic bone disorders, although their correlation has not yet been fully understood. VDR is also implicated in the renal and intestinal absorption of calcium and phosphate, as well as the downregulation of PTH synthesis, and it appears to play a role in the management of persistent secondary hyperparathyroidism after renal transplantation.

Beyond the role of VD transporter, VDBP also has immunological and anti-inflammatory properties. This happens, for example, in the context of multiple sclerosis, where the variation in VDBP levels, in synergy with VD insufficiency, would negatively influence the severity and duration of the disease [3].

VD is also involved in fighting infectious processes, where it promotes the synthesis of antimicrobial peptides, suggesting a likely mechanism by which this molecule could provide protection against some respiratory pathogens, including severe acute respiratory syndrome coronavirus-2, although current evidence is inconsistent regarding its effect on the outcomes and length of hospital stay of such patients [4]. Notably, VD deficiency favors the chronicity of hepatitis B virus infection and, consequently, the degree of fibrosis and the progression to liver cirrhosis.

Finally, VD plays a fundamental and widely demonstrated role in the prevention of oxidative stress. Indeed, its deficiency induces the interruption of mitochondrial function and is associated with markers of inflammation. Furthermore, the conversion of 25(OH)D3 into 1,25(OH)2D3 occurs at the extrarenal tissue level by stimulating the production of macrophages through the type 2 IFN response. This condition is particularly expressed in the context of human exposure to organochlorine pesticides, potentially capable of determining the interruption of the conversion of 25(OH)D3 up to ’1,25(OH)2D3. Interference with the function of VDRs in the body and impairment of calcium homeostasis results in VD deficiency and increased risk of developing chronic conditions related to increased oxidative stress and chronic inflammation, such as diabetes and cardiovascular diseases [5]. The same mechanism is also found in the context of polycystic ovary syndrome, where VD integration may be able to reduce the levels of oxidative stress involved in the pathogenetic mechanisms of this pathology in synergy with other chronic pathologies.
